# Diethyl 2-[phen­yl(pyrazol-1-yl)meth­yl]propane­dioate

**DOI:** 10.1107/S1600536810011748

**Published:** 2010-04-02

**Authors:** Ihssan Meskini, Maria Daoudi, Jean-Claude Daran, Hafid Zouihri, Taibi Ben Hadda

**Affiliations:** a Laboratoire de Chimie Organique, Faculté des Sciences Dhar el Mahraz, Université Sidi Mohammed Ben Abdellah, Fès, Morocco; b Laboratoire de Chimie de Coordination, 205 Route de Narbonne, 31077 Toulouse Cedex, France; c Centre National pour la Recherche Scientifique et Technique, Division UATRS, Rabat, Morocco; d Laboratoire de Chimie des Matériaux, Université Med. 1ier, Oujda, Morocco

## Abstract

There are two independent mol­ecules in the asymmetric unit of the title compound, C_17_H_20_N_2_O_4_, which differ slightly in the orientation of the phenyl ring and carbonyl groups with respect to the pyrazole unit. In the first mol­ecule, the dihedral angle between the phenyl and pyrazole rings is 68.99 (13)° while the two carbonyl groups make a dihedral angle of 72.1 (4)°. The corresponding values in the second mol­ecule are 68.54 (14) and 71.5 (4)°, respectively.

## Related literature

For related compounds displaying biological activity, see: Dayam *et al.* (2007 ▶); Patil *et al.* (2007 ▶); Ramkumar *et al.* (2008 ▶); Sechi *et al.* (2009*a* ▶,*b* ▶); Zeng *et al.* (2008*a* ▶,*b* ▶). For a related structures, see: Akkurt *et al.* (2007 ▶). For the synthetic procedure, see: Pommier & Neamati (2006 ▶). For bond-length data, see: Allen *et al.* (1987 ▶).
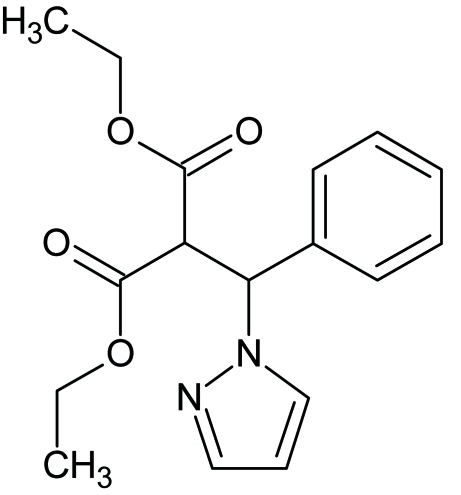


## Experimental

### 

#### Crystal data

C_17_H_20_N_2_O_4_
*M* *_r_* = 316.36
Monoclinic, 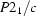

*a* = 19.6279 (8) Å
*b* = 8.1538 (3) Å
*c* = 21.6002 (9) Å
β = 104.675 (2)°
*V* = 3344.2 (2) Å^3^
*Z* = 8
Mo *K*α radiation
μ = 0.09 mm^−1^
*T* = 173 K
0.35 × 0.22 × 0.17 mm

#### Data collection

Bruker X8 APEXII CCD area-detector diffractometer
34259 measured reflections
6348 independent reflections
4175 reflections with *I* > 2σ(*I*)
*R*_int_ = 0.048

#### Refinement

*R*[*F* ^2^ > 2σ(*F* ^2^)] = 0.043
*wR*(*F* ^2^) = 0.122
*S* = 1.06
6346 reflections
419 parameters
H-atom parameters constrained
Δρ_max_ = 0.19 e Å^−3^
Δρ_min_ = −0.25 e Å^−3^

### 

Data collection: *APEX2* (Bruker, 2005 ▶); cell refinement: *SAINT* (Bruker, 2005 ▶); data reduction: *SAINT*; program(s) used to solve structure: *SHELXS97* (Sheldrick, 2008 ▶); program(s) used to refine structure: *SHELXL97* (Sheldrick, 2008 ▶); molecular graphics: *PLATON* (Spek, 2009 ▶); software used to prepare material for publication: *publCIF* (Westrip, 2010 ▶).

## Supplementary Material

Crystal structure: contains datablocks I, global. DOI: 10.1107/S1600536810011748/kj2142sup1.cif

Structure factors: contains datablocks I. DOI: 10.1107/S1600536810011748/kj2142Isup2.hkl

Additional supplementary materials:  crystallographic information; 3D view; checkCIF report

## Figures and Tables

**Fig. 1.**
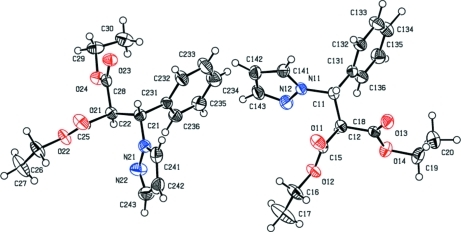
Molecular structure of the title compound with the atom-labelling scheme. Displacement ellipsoids are drawn at the 50% probability level.

**Fig. 2.**
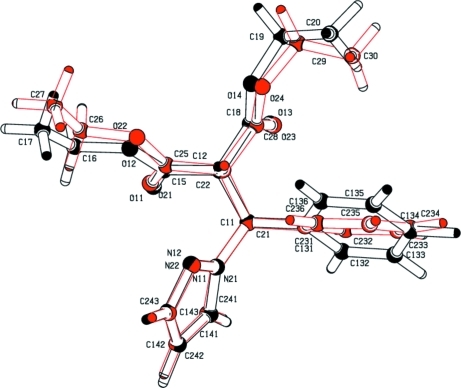
View showing the fitting between the molecule 1 and the inverted molecule 2 from the asymmetric unit [PLATON (Spek, 2009)].

## supplementary crystallographic information

### Comment

The rational design of new HIV-1 Integrase (H-I) inhibitors, one validated target for chemotherapeutic intervention (Dayam *et al.*, 2007), is fundamentally based on intermolecular coordination between H-I / chemical inhibitor / metals (Mg^+2^ and Mn^+2^, co-factors of the enzyme), leading to the formation of bimetallic complexes (Zeng *et al.*, 2008a; Sechi *et al.*, 2009a). Therefore several bimetallic metal complexes, in many cases exploring the well-known polydentate ligands, appear in this scenario as the most promising concept to employ in either enzyme / drug interaction or electron transfer process, in the last case involving biological oxygen transfer (Sechi *et al.*, 2009b; Ramkumar *et al.*, 2008). Another exciting example of the application of such polydentate ligands involves the synergic water activation, that occurs via the so-called remote metallic atoms. Such organometallic compounds are expected to promote or block the H-I activity [Zeng *et al.* (2008b)]. The examples given above clearly demonstrate that polydentate ligands are of special interest in the field of bioorganometallic chemistry [Patil *et al.* (2007)].

The structure of the title compound was established by ^1^H and ^13^C NMR and confirmed by its elemental analyses and single-crystal X-ray structure. Crystals of the title compound contain two molecules in the asymmetric unit. The difference between the molecules lies in the orientation of the phenyl and pyrazol rings and carbonyl planes in each molecule as shown in the fitting drawing (Fig. 2). Thus in the first molecule (C11 to C143) the dihedral angles between the phenyl and pyrazol rings is 68.99 (13)° and between the two carbonyl groups is 72.1 (4)°. Whereas in the second molecule (C21 to C243), equivalent angles have as values 68.54 (14)° and 71.5 (4)°, respectively. The conformational difference between the independent molecules, as shown in Fig. 2, can also be described by torsion angles: N11—C11···C131—C132 = 79.71 (15), C11—C12···O11O12 = 46.54 (9) and C11—C12···O13—O14 = 47.02 (9) in the first molecule. In the second molecule, the corresponding values are 54.31 (14), 41.77 (9) and 47.44 (9), respectively. The bond lengths and angles in the title compound (Fig. 1) are found to have normal values [Allen *et al.*, 1987].

### Experimental

To a solution of diethyl benzylpropanedioate (5 mmol) in water (25 ml) was added 1*H*-pyrazol (6 mmol) in the presence of acetic acid (0.1% mol). The mixture was stirred continuously at room temperature until the starting material was completely consumed. After removing the solvent, the crude products were dissolved in diethyl ether (2 x 40 ml) and washed with water until the pH became neutral. The organic solvent was dried with sodium sulphate and then evaporated. The residue was purified by recrystallization from a mixture ether/hexane (1:1) to give a white solid in 74% yield. R_f_ = 0.45 (ether/hexane: 1/1). Elemental analysis for C_17_H_20_N_2_O_4_: Calcd (Found): C 67.82 (67.79), H 5.89 (5.87), N (2.73 (2.72). The purity of the compound was checked by determining its melting point (87-89°C). Suitable single crystal of the title compound were obtained by recrystallization from ethanol.

### Refinement

All H atoms were fixed geometrically and treated as riding with C—H = 0.95 Å (aromatic), 0.99 Å (methylene), 0.98 Å (methyl) and 1.00 Å (methine) with Uiso(H) = 1.2Ueq (aromatic, methine, methylene) and Uiso(H) = 1.5Ueq (methyl).

### Crystal data

**Table:**

C_17_H_20_N_2_O_4_	*F*(000) = 1344
*M**_r_* = 316.36	*D*_x_ = 1.257 Mg m^−^^3^
Monoclinic, *P*2_1_/*c*	Melting point: 360 K
Hall symbol: -P 2ybc	Mo *K*α radiation, λ = 0.71073 Å
*a* = 19.6279 (8) Å	Cell parameters from 3174 reflections
*b* = 8.1538 (3) Å	θ = 2.1–25.2°
*c* = 21.6002 (9) Å	µ = 0.09 mm^−^^1^
β = 104.675 (2)°	*T* = 173 K
*V* = 3344.2 (2) Å^3^	Block, colourless
*Z* = 8	0.35 × 0.22 × 0.17 mm

### Data collection

**Table:**

Bruker X8 APEXII CCD area-detector diffractometer	4175 reflections with *I* > 2σ(*I*)
Radiation source: fine-focus sealed tube	*R*_int_ = 0.048
graphite	θ_max_ = 25.7°, θ_min_ = 1.1°
φ and ω scans	*h* = −23→23
34259 measured reflections	*k* = −9→9
6348 independent reflections	*l* = −26→23

### Refinement

**Table:**

Refinement on *F*^2^	Primary atom site location: structure-invariant direct methods
Least-squares matrix: full	Secondary atom site location: difference Fourier map
*R*[*F*^2^ > 2σ(*F*^2^)] = 0.043	Hydrogen site location: inferred from neighbouring sites
*wR*(*F*^2^) = 0.122	H-atom parameters constrained
*S* = 1.06	*w* = 1/[σ^2^(*F*_o_^2^) + (0.0532*P*)^2^ + 0.7393*P*] where *P* = (*F*_o_^2^ + 2*F*_c_^2^)/3
6346 reflections	(Δ/σ)_max_ = 0.001
419 parameters	Δρ_max_ = 0.19 e Å^−^^3^
0 restraints	Δρ_min_ = −0.25 e Å^−^^3^

### Special details

**Table:**

Experimental. The crystal structure was confirmed by elemental analysis and 1H and 13 C-NMR.IR (KBr) ν cm-1 : 2896/2985 (CH), 1748 (CO), 1514/1595 (C=C), 1292/1308 (C—O), 1175, 1139, 1013, 866, 753, 440.^1^H-NMR (250 MHz, CDCl_3_) d (ppm): 7.30-7.46 (m, ^4^H, aromat, ^3^J = 8.35 Hz), 6.20 (t, ^1^H, C^4^Pz, ^3^J = 2 Hz), 7.5 (d, 2H, C^3'^H and C^5^HPz, ^3^J = 14.4 Hz), 5.85 (d, 1H, PhC^3^H, ^3^J = 11.36 Hz), 4.80 (d, 1H, C^2^H(CO_2_Et)_2_, ^3^J = 11.11 Hz), 3.95 (dq, 2 H_AB_, OCH_2_CH_3_,J_AB_= 14.30 Hz, ^3^J = 7.11 Hz), 4,12 (dq, 2H_AB_, CH_2_OCH_3_, J_AB_= 14.30 Hz, ^3^J = 7.11 Hz), 1.15 (t, 3H, OCH_2_CH_3_ , ^3^J = 7.13 Hz), 1.01 (t, 3H, OCH_2_CH_3_, ^3^J = 7.13 Hz).^13^C-NMR (250 MHz, CDCl_3_) δ (ppm): 166.37 (C=O), 166.61 (CO), 137.15 (C_quat_, Ph), 128,62 (C_tert_, 2Cmeta/arm, Ph), 129.76 (C_tert_, 2Cortho/ arm, Ph), 139.56 (C_tert_,' C^5'^'Pz), 128.67 (C_tert_, '^C3'^'Pz), 105.71 (C_tert_, C^4^H, Pz), 61.87/ 61.76 (C_sec_, 2CH_2_, ester), 64.22 (C_tert_, C^3^HPh), 57.33 (C_tert_, C^2^H(CO_2_Et)_2_), 13.87 (C, OCH_2_CH_3_, ester), 13.69 (C, OCH_2_CH_3_, ester).MS (IE) Calcd for [M]^+^ C_17_H_20_N_2_O_4_: 316.35, [M+H]^+^. = 317, [M - CH(CO_2_Et)_2_]^+^. = 157 (100%).Elemental analysis for C_17_H_20_N_2_O_4_ Calcd (Found): C 64.54 (64.37), H 6.37 (6.34), N 8.86 (8.84).
Geometry. All esds (except the esd in the dihedral angle between two l.s. planes) are estimated using the full covariance matrix. The cell esds are taken into account individually in the estimation of esds in distances, angles and torsion angles; correlations between esds in cell parameters are only used when they are defined by crystal symmetry. An approximate (isotropic) treatment of cell esds is used for estimating esds involving l.s. planes.
Refinement. Refinement of F^2^ against ALL reflections. The weighted R-factor wR and goodness of fit S are based on F^2^, conventional R-factors R are based on F, with F set to zero for negative F^2^. The threshold expression of F^2^ > 2sigma(F^2^) is used only for calculating R-factors(gt) etc. and is not relevant to the choice of reflections for refinement. R-factors based on F^2^ are statistically about twice as large as those based on F, and R- factors based on ALL data will be even larger.

### Fractional atomic coordinates and isotropic or equivalent isotropic displacement parameters (Å^2^)

**Table:**

	*x*	*y*	*z*	*U*_iso_*/*U*_eq_	
C11	0.97782 (9)	0.4587 (2)	0.14742 (9)	0.0288 (4)	
H11	0.9766	0.4313	0.1922	0.035*	
C12	0.94763 (9)	0.3117 (2)	0.10526 (9)	0.0285 (4)	
H12	0.9474	0.3357	0.0598	0.034*	
C136	1.08085 (10)	0.4753 (2)	0.09490 (10)	0.0355 (5)	
H136	1.0511	0.4353	0.0561	0.043*	
C131	1.05405 (9)	0.4968 (2)	0.14811 (9)	0.0293 (4)	
C132	1.09818 (10)	0.5576 (3)	0.20416 (10)	0.0377 (5)	
H132	1.0805	0.5740	0.2408	0.045*	
C133	1.16781 (11)	0.5945 (3)	0.20699 (11)	0.0462 (6)	
H133	1.1975	0.6370	0.2454	0.055*	
C135	1.15052 (11)	0.5120 (3)	0.09818 (11)	0.0432 (5)	
H135	1.1683	0.4972	0.0616	0.052*	
C134	1.19419 (11)	0.5697 (3)	0.15430 (11)	0.0478 (6)	
H134	1.2423	0.5924	0.1567	0.057*	
C18	0.99255 (9)	0.1608 (2)	0.12819 (9)	0.0302 (4)	
C15	0.87274 (9)	0.2760 (2)	0.10988 (9)	0.0301 (4)	
C16	0.75841 (10)	0.2018 (3)	0.05069 (10)	0.0478 (6)	
H16A	0.7385	0.2926	0.0710	0.057*	
H16B	0.7540	0.0989	0.0737	0.057*	
C19	1.04535 (12)	−0.0718 (3)	0.09243 (11)	0.0462 (6)	
H19A	1.0208	−0.1668	0.0681	0.055*	
H19B	1.0561	−0.0982	0.1386	0.055*	
C20	1.11176 (11)	−0.0376 (3)	0.07335 (11)	0.0484 (6)	
H20A	1.1007	−0.0113	0.0276	0.073*	
H20B	1.1422	−0.1346	0.0818	0.073*	
H20C	1.1361	0.0555	0.0981	0.073*	
C17	0.72063 (12)	0.1870 (5)	−0.01707 (12)	0.0850 (11)	
H17A	0.7247	0.2901	−0.0392	0.127*	
H17B	0.6708	0.1635	−0.0205	0.127*	
H17C	0.7412	0.0976	−0.0367	0.127*	
O13	1.01617 (7)	0.12639 (17)	0.18357 (7)	0.0448 (4)	
O14	1.00073 (7)	0.07390 (16)	0.07883 (6)	0.0390 (3)	
O12	0.83217 (6)	0.23431 (18)	0.05337 (6)	0.0388 (3)	
O11	0.85427 (7)	0.28256 (18)	0.15869 (6)	0.0420 (4)	
N11	0.93182 (8)	0.60116 (18)	0.12721 (7)	0.0295 (4)	
C21	0.47116 (9)	0.8377 (2)	0.40329 (9)	0.0294 (4)	
H21	0.4728	0.8760	0.4476	0.035*	
C22	0.43696 (9)	0.9732 (2)	0.35641 (9)	0.0282 (4)	
H22	0.4362	0.9388	0.3118	0.034*	
C236	0.56098 (10)	0.7580 (2)	0.34270 (9)	0.0357 (5)	
H236	0.5236	0.7390	0.3058	0.043*	
C231	0.54615 (9)	0.8070 (2)	0.39927 (8)	0.0286 (4)	
C235	0.62987 (11)	0.7365 (3)	0.33975 (10)	0.0422 (5)	
H235	0.6397	0.7029	0.3008	0.051*	
C232	0.60142 (10)	0.8345 (3)	0.45220 (10)	0.0435 (5)	
H232	0.5920	0.8683	0.4913	0.052*	
C233	0.67022 (11)	0.8134 (3)	0.44898 (12)	0.0556 (6)	
H233	0.7077	0.8335	0.4857	0.067*	
C234	0.68458 (11)	0.7637 (3)	0.39325 (11)	0.0504 (6)	
H234	0.7319	0.7480	0.3913	0.060*	
O22	0.32221 (6)	1.05521 (18)	0.30375 (6)	0.0379 (3)	
O23	0.50629 (7)	1.17430 (17)	0.42706 (6)	0.0383 (3)	
O24	0.48859 (7)	1.20201 (17)	0.32046 (6)	0.0374 (3)	
O21	0.34248 (7)	1.00089 (19)	0.40842 (6)	0.0428 (4)	
C25	0.36234 (10)	1.0097 (2)	0.36044 (9)	0.0303 (4)	
C26	0.24856 (10)	1.0901 (3)	0.30130 (10)	0.0483 (6)	
H26A	0.2450	1.1902	0.3262	0.058*	
H26B	0.2274	0.9975	0.3195	0.058*	
C27	0.21148 (12)	1.1144 (4)	0.23293 (11)	0.0702 (8)	
H27A	0.2300	1.2124	0.2166	0.105*	
H27B	0.1609	1.1282	0.2289	0.105*	
H27C	0.2190	1.0184	0.2081	0.105*	
C28	0.48128 (9)	1.1279 (2)	0.37332 (9)	0.0285 (4)	
C29	0.53627 (11)	1.3421 (3)	0.32954 (10)	0.0439 (5)	
H29A	0.5384	1.3949	0.3713	0.053*	
H29B	0.5190	1.4239	0.2953	0.053*	
C30	0.60821 (11)	1.2832 (3)	0.32758 (11)	0.0526 (6)	
H30A	0.6245	1.2005	0.3610	0.079*	
H30B	0.6410	1.3760	0.3349	0.079*	
H30C	0.6060	1.2348	0.2856	0.079*	
N21	0.42781 (8)	0.68893 (19)	0.39088 (7)	0.0323 (4)	
N12	0.90130 (9)	0.6301 (2)	0.06472 (8)	0.0402 (4)	
N22	0.39520 (10)	0.6437 (2)	0.33069 (8)	0.0469 (5)	
C141	0.91609 (10)	0.7177 (2)	0.16551 (10)	0.0350 (5)	
H141	0.9323	0.7224	0.2108	0.042*	
C241	0.41790 (11)	0.5804 (3)	0.43464 (11)	0.0412 (5)	
H241	0.4364	0.5869	0.4797	0.049*	
C142	0.87256 (10)	0.8278 (3)	0.12717 (10)	0.0394 (5)	
H142	0.8520	0.9236	0.1398	0.047*	
C143	0.86510 (10)	0.7683 (3)	0.06560 (11)	0.0431 (5)	
H143	0.8374	0.8202	0.0283	0.052*	
C242	0.37550 (11)	0.4572 (3)	0.40125 (12)	0.0480 (6)	
H242	0.3586	0.3625	0.4183	0.058*	
C243	0.36321 (12)	0.5025 (3)	0.33775 (12)	0.0487 (6)	
H243	0.3354	0.4407	0.3032	0.058*	

### Atomic displacement parameters (Å^2^)

**Table:**

	*U*^11^	*U*^22^	*U*^33^	*U*^12^	*U*^13^	*U*^23^
C11	0.0257 (10)	0.0313 (10)	0.0280 (10)	0.0028 (8)	0.0042 (8)	0.0022 (8)
C12	0.0242 (10)	0.0341 (11)	0.0268 (10)	0.0018 (8)	0.0056 (7)	0.0009 (8)
C136	0.0305 (11)	0.0385 (12)	0.0368 (12)	−0.0014 (9)	0.0072 (9)	−0.0019 (9)
C131	0.0255 (10)	0.0282 (10)	0.0334 (11)	0.0029 (8)	0.0059 (8)	0.0019 (8)
C132	0.0334 (11)	0.0435 (12)	0.0346 (12)	0.0013 (9)	0.0054 (9)	−0.0015 (9)
C133	0.0326 (12)	0.0541 (14)	0.0464 (14)	−0.0053 (10)	0.0004 (10)	−0.0046 (11)
C135	0.0360 (12)	0.0500 (13)	0.0466 (14)	−0.0007 (10)	0.0158 (10)	0.0005 (11)
C134	0.0284 (11)	0.0575 (14)	0.0563 (15)	−0.0015 (11)	0.0085 (10)	0.0011 (12)
C18	0.0222 (10)	0.0327 (11)	0.0344 (12)	−0.0039 (8)	0.0046 (8)	−0.0005 (9)
C15	0.0270 (10)	0.0297 (10)	0.0323 (11)	0.0049 (8)	0.0049 (8)	0.0015 (8)
C16	0.0221 (10)	0.0735 (16)	0.0472 (14)	−0.0042 (11)	0.0076 (9)	−0.0031 (12)
C19	0.0516 (14)	0.0332 (12)	0.0600 (15)	0.0111 (10)	0.0252 (11)	0.0019 (10)
C20	0.0404 (13)	0.0569 (15)	0.0483 (14)	0.0113 (11)	0.0121 (10)	0.0028 (11)
C17	0.0348 (14)	0.166 (3)	0.0485 (16)	−0.0270 (17)	0.0003 (11)	0.0012 (18)
O13	0.0481 (9)	0.0433 (9)	0.0355 (9)	0.0110 (7)	−0.0031 (7)	−0.0010 (7)
O14	0.0448 (8)	0.0343 (8)	0.0409 (8)	0.0087 (7)	0.0165 (7)	0.0012 (6)
O12	0.0229 (7)	0.0566 (9)	0.0352 (8)	−0.0039 (6)	0.0041 (6)	−0.0065 (7)
O11	0.0327 (8)	0.0603 (10)	0.0335 (8)	−0.0014 (7)	0.0094 (6)	−0.0001 (7)
N11	0.0252 (8)	0.0317 (9)	0.0299 (9)	0.0017 (7)	0.0039 (7)	0.0021 (7)
C21	0.0289 (10)	0.0337 (10)	0.0245 (10)	−0.0001 (8)	0.0049 (8)	−0.0016 (8)
C22	0.0268 (10)	0.0341 (11)	0.0236 (10)	0.0003 (8)	0.0059 (8)	0.0000 (8)
C236	0.0344 (11)	0.0405 (11)	0.0310 (11)	−0.0016 (9)	0.0062 (8)	−0.0013 (9)
C231	0.0288 (10)	0.0288 (10)	0.0272 (10)	0.0001 (8)	0.0054 (8)	0.0012 (8)
C235	0.0415 (12)	0.0468 (13)	0.0411 (13)	−0.0005 (10)	0.0158 (10)	−0.0022 (10)
C232	0.0346 (12)	0.0580 (14)	0.0346 (12)	0.0077 (10)	0.0026 (9)	−0.0046 (10)
C233	0.0344 (13)	0.0740 (17)	0.0524 (15)	0.0060 (12)	0.0000 (10)	−0.0078 (13)
C234	0.0344 (12)	0.0590 (15)	0.0581 (16)	0.0001 (11)	0.0122 (11)	−0.0034 (12)
O22	0.0224 (7)	0.0570 (9)	0.0334 (8)	0.0031 (6)	0.0054 (6)	0.0090 (7)
O23	0.0419 (8)	0.0419 (8)	0.0287 (8)	−0.0041 (7)	0.0043 (6)	−0.0038 (6)
O24	0.0402 (8)	0.0416 (8)	0.0302 (8)	−0.0081 (6)	0.0082 (6)	0.0030 (6)
O21	0.0324 (8)	0.0674 (10)	0.0299 (8)	−0.0005 (7)	0.0104 (6)	−0.0010 (7)
C25	0.0277 (10)	0.0327 (11)	0.0285 (11)	−0.0040 (8)	0.0037 (8)	−0.0022 (8)
C26	0.0202 (10)	0.0755 (17)	0.0489 (14)	0.0020 (11)	0.0081 (9)	0.0093 (12)
C27	0.0298 (12)	0.125 (3)	0.0508 (15)	0.0155 (15)	0.0017 (11)	0.0139 (16)
C28	0.0241 (10)	0.0337 (11)	0.0272 (11)	0.0053 (8)	0.0055 (8)	0.0006 (8)
C29	0.0505 (13)	0.0409 (12)	0.0404 (13)	−0.0152 (11)	0.0116 (10)	0.0010 (10)
C30	0.0447 (14)	0.0648 (16)	0.0469 (14)	−0.0156 (12)	0.0088 (10)	−0.0025 (12)
N21	0.0313 (9)	0.0345 (9)	0.0312 (9)	−0.0002 (7)	0.0080 (7)	0.0012 (7)
N12	0.0418 (10)	0.0415 (10)	0.0327 (10)	0.0035 (8)	0.0011 (8)	0.0037 (8)
N22	0.0532 (12)	0.0470 (11)	0.0371 (11)	−0.0123 (9)	0.0054 (8)	−0.0015 (8)
C141	0.0332 (11)	0.0358 (11)	0.0373 (12)	0.0013 (9)	0.0111 (9)	−0.0025 (9)
C241	0.0372 (12)	0.0429 (13)	0.0463 (13)	0.0082 (10)	0.0157 (10)	0.0133 (10)
C142	0.0320 (11)	0.0353 (11)	0.0520 (14)	0.0042 (9)	0.0127 (9)	0.0025 (10)
C143	0.0322 (11)	0.0430 (13)	0.0483 (14)	0.0029 (10)	−0.0005 (9)	0.0134 (10)
C242	0.0415 (13)	0.0367 (13)	0.0712 (17)	0.0041 (10)	0.0245 (12)	0.0092 (11)
C243	0.0461 (13)	0.0410 (13)	0.0569 (16)	−0.0096 (11)	0.0090 (11)	−0.0048 (11)

### Geometric parameters (Å, °)

**Table:**

C11—N11	1.468 (2)	C22—H22	1.0000
C11—C131	1.525 (2)	C236—C235	1.381 (3)
C11—C12	1.531 (3)	C236—C231	1.385 (3)
C11—H11	1.0000	C236—H236	0.9500
C12—C18	1.522 (3)	C231—C232	1.381 (3)
C12—C15	1.526 (2)	C235—C234	1.382 (3)
C12—H12	1.0000	C235—H235	0.9500
C136—C135	1.384 (3)	C232—C233	1.380 (3)
C136—C131	1.391 (3)	C232—H232	0.9500
C136—H136	0.9500	C233—C234	1.365 (3)
C131—C132	1.390 (3)	C233—H233	0.9500
C132—C133	1.386 (3)	C234—H234	0.9500
C132—H132	0.9500	O22—C25	1.330 (2)
C133—C134	1.379 (3)	O22—C26	1.461 (2)
C133—H133	0.9500	O23—C28	1.201 (2)
C135—C134	1.378 (3)	O24—C28	1.331 (2)
C135—H135	0.9500	O24—C29	1.458 (2)
C134—H134	0.9500	O21—C25	1.198 (2)
C18—O13	1.202 (2)	C26—C27	1.485 (3)
C18—O14	1.323 (2)	C26—H26A	0.9900
C15—O11	1.200 (2)	C26—H26B	0.9900
C15—O12	1.322 (2)	C27—H27A	0.9800
C16—O12	1.459 (2)	C27—H27B	0.9800
C16—C17	1.469 (3)	C27—H27C	0.9800
C16—H16A	0.9900	C29—C30	1.502 (3)
C16—H16B	0.9900	C29—H29A	0.9900
C19—O14	1.461 (2)	C29—H29B	0.9900
C19—C20	1.489 (3)	C30—H30A	0.9800
C19—H19A	0.9900	C30—H30B	0.9800
C19—H19B	0.9900	C30—H30C	0.9800
C20—H20A	0.9800	N21—C241	1.345 (2)
C20—H20B	0.9800	N21—N22	1.347 (2)
C20—H20C	0.9800	N12—C143	1.335 (3)
C17—H17A	0.9800	N22—C243	1.339 (3)
C17—H17B	0.9800	C141—C142	1.365 (3)
C17—H17C	0.9800	C141—H141	0.9500
N11—C141	1.346 (2)	C241—C242	1.385 (3)
N11—N12	1.352 (2)	C241—H241	0.9500
C21—N21	1.467 (2)	C142—C143	1.388 (3)
C21—C231	1.517 (2)	C142—H142	0.9500
C21—C22	1.534 (2)	C143—H143	0.9500
C21—H21	1.0000	C242—C243	1.382 (3)
C22—C25	1.519 (2)	C242—H242	0.9500
C22—C28	1.524 (3)	C243—H243	0.9500
			
N11—C11—C131	111.71 (15)	C28—C22—H22	109.4
N11—C11—C12	109.05 (14)	C21—C22—H22	109.4
C131—C11—C12	113.35 (15)	C235—C236—C231	120.30 (18)
N11—C11—H11	107.5	C235—C236—H236	119.9
C131—C11—H11	107.5	C231—C236—H236	119.9
C12—C11—H11	107.5	C232—C231—C236	118.74 (18)
C18—C12—C15	108.20 (15)	C232—C231—C21	119.74 (17)
C18—C12—C11	109.54 (14)	C236—C231—C21	121.46 (16)
C15—C12—C11	110.16 (15)	C236—C235—C234	120.2 (2)
C18—C12—H12	109.6	C236—C235—H235	119.9
C15—C12—H12	109.6	C234—C235—H235	119.9
C11—C12—H12	109.6	C233—C232—C231	120.8 (2)
C135—C136—C131	120.42 (19)	C233—C232—H232	119.6
C135—C136—H136	119.8	C231—C232—H232	119.6
C131—C136—H136	119.8	C234—C233—C232	120.3 (2)
C132—C131—C136	118.83 (17)	C234—C233—H233	119.9
C132—C131—C11	118.35 (17)	C232—C233—H233	119.9
C136—C131—C11	122.82 (17)	C233—C234—C235	119.6 (2)
C133—C132—C131	120.40 (19)	C233—C234—H234	120.2
C133—C132—H132	119.8	C235—C234—H234	120.2
C131—C132—H132	119.8	C25—O22—C26	115.94 (14)
C134—C133—C132	120.3 (2)	C28—O24—C29	116.43 (15)
C134—C133—H133	119.9	O21—C25—O22	124.50 (17)
C132—C133—H133	119.9	O21—C25—C22	124.60 (17)
C134—C135—C136	120.3 (2)	O22—C25—C22	110.90 (15)
C134—C135—H135	119.8	O22—C26—C27	107.04 (16)
C136—C135—H135	119.8	O22—C26—H26A	110.3
C135—C134—C133	119.7 (2)	C27—C26—H26A	110.3
C135—C134—H134	120.1	O22—C26—H26B	110.3
C133—C134—H134	120.1	C27—C26—H26B	110.3
O13—C18—O14	125.59 (18)	H26A—C26—H26B	108.6
O13—C18—C12	123.95 (17)	C26—C27—H27A	109.5
O14—C18—C12	110.45 (16)	C26—C27—H27B	109.5
O11—C15—O12	125.28 (18)	H27A—C27—H27B	109.5
O11—C15—C12	124.01 (17)	C26—C27—H27C	109.5
O12—C15—C12	110.70 (16)	H27A—C27—H27C	109.5
O12—C16—C17	107.46 (17)	H27B—C27—H27C	109.5
O12—C16—H16A	110.2	O23—C28—O24	125.42 (18)
C17—C16—H16A	110.2	O23—C28—C22	124.14 (17)
O12—C16—H16B	110.2	O24—C28—C22	110.44 (15)
C17—C16—H16B	110.2	O24—C29—C30	108.77 (18)
H16A—C16—H16B	108.5	O24—C29—H29A	109.9
O14—C19—C20	108.32 (17)	C30—C29—H29A	109.9
O14—C19—H19A	110.0	O24—C29—H29B	109.9
C20—C19—H19A	110.0	C30—C29—H29B	109.9
O14—C19—H19B	110.0	H29A—C29—H29B	108.3
C20—C19—H19B	110.0	C29—C30—H30A	109.5
H19A—C19—H19B	108.4	C29—C30—H30B	109.5
C19—C20—H20A	109.5	H30A—C30—H30B	109.5
C19—C20—H20B	109.5	C29—C30—H30C	109.5
H20A—C20—H20B	109.5	H30A—C30—H30C	109.5
C19—C20—H20C	109.5	H30B—C30—H30C	109.5
H20A—C20—H20C	109.5	C241—N21—N22	112.35 (17)
H20B—C20—H20C	109.5	C241—N21—C21	126.61 (17)
C16—C17—H17A	109.5	N22—N21—C21	120.98 (15)
C16—C17—H17B	109.5	C143—N12—N11	103.65 (16)
H17A—C17—H17B	109.5	C243—N22—N21	104.33 (17)
C16—C17—H17C	109.5	N11—C141—C142	107.29 (18)
H17A—C17—H17C	109.5	N11—C141—H141	126.4
H17B—C17—H17C	109.5	C142—C141—H141	126.4
C18—O14—C19	117.54 (16)	N21—C241—C242	106.64 (19)
C15—O12—C16	116.30 (15)	N21—C241—H241	126.7
C141—N11—N12	112.20 (16)	C242—C241—H241	126.7
C141—N11—C11	126.54 (16)	C141—C142—C143	104.45 (18)
N12—N11—C11	121.24 (15)	C141—C142—H142	127.8
N21—C21—C231	112.30 (15)	C143—C142—H142	127.8
N21—C21—C22	109.77 (14)	N12—C143—C142	112.41 (18)
C231—C21—C22	110.48 (15)	N12—C143—H143	123.8
N21—C21—H21	108.1	C142—C143—H143	123.8
C231—C21—H21	108.1	C243—C242—C241	104.70 (19)
C22—C21—H21	108.1	C243—C242—H242	127.7
C25—C22—C28	108.93 (15)	C241—C242—H242	127.7
C25—C22—C21	111.74 (15)	N22—C243—C242	112.0 (2)
C28—C22—C21	108.08 (15)	N22—C243—H243	124.0
C25—C22—H22	109.4	C242—C243—H243	124.0
